# Implementation of nursing process in clinical settings: the case of three governmental hospitals in Ethiopia, 2017

**DOI:** 10.1186/s13104-018-3275-z

**Published:** 2018-03-13

**Authors:** Ayele Semachew

**Affiliations:** Department of Nursing, College of Medicine and Health Sciences, Bahir Dar University, PO Box. 79, Bahir Dar, Ethiopia

**Keywords:** Nursing, Nursing process, Implementation, Survey, North West Ethiopia

## Abstract

**Objective:**

The purpose of this survey was to evaluate the implementation of the nursing process at three randomly selected governmental hospitals found in Amhara Region North West Ethiopia.

**Result:**

From the total 338 reviewed documents, 264 (78.1%) have a nursing process format attached with the patient’s profile/file, 107 (31.7%) had no nursing diagnosis, 185 (54.7%) of nurses stated their plan of care based on priority, 173 (51.2%) of nurses did not document their interventions based on plan and 179 (53.0%) of nurses did not evaluate their interventions. The overall implementation of nursing process among Felege Hiwot Referal hospital, Debretabor general hospital and Finoteselam general hospitals were 49.12, 68.18, and 69.42% respectively. Nursing professionals shall improve documentation required in implementing the nursing process. Nursing managers (matron, ward heads) shall supervise the overall implementation of nursing process. Hospital nursing services managers (matrons) shall arrange and facilitate case presentations by the nursing staffs which focus on documentation and updates on nursing process. Hospitals need to establish and support nursing process coordinating staff in their institution.

## Introduction

Nursing has always been directed to keep people healthy and provide comfort, care, and assurance to the patients. The nursing care, therefore, may involve any number of activities ranging from carrying out complicated technical procedures to something as simple as holding a hand of the patient. The central focus of nursing care is the person receiving care and included the physical, emotional, social and spiritual dimensions of that person. Nursing care, therefore, refers to care of others [1].

Nurses have many demands on their time as they provide care and document their work in a descriptive manner. In order to better meet the needs of their patients, the nurses developed a care plan with special forms [2].

Nursing process (NP) is a systematic method which utilizes scientific reasoning, problem-solving and critical thinking to direct nurses in caring for patients effectively [3, 4].

The nursing process is a systematic problem-solving approach used to identify, prevent and treat actual or potential health problems and promote wellness. It has five steps; Assessment, Diagnosis, planning, implementation and evaluation [5].

The nursing process was initially an adapted form of problem-solving technique based on theory used by nurses every day to help patients improve their health and assist doctors in treating patients. Its primary aim is to know the health status and the problems of clients which may be actual or potential. It is made up of a series of stages that are used to achieve the objective—the health improvement of the patient. The use of nursing process can stop at any stage as deemed necessary or can be repeated as needed. This process is inclusive of physical health as well as the emotional aspects of patient health. Nursing knowledge is used throughout the process to formulate changes in approach to the patient’s changing condition [6].

Many nurse researchers and theorists are in agreement that nursing process is a scientific method for delivering holistic and quality nursing care. Therefore, its effective implementation is critical for improved quality of nursing care. When the quality of nursing care is improved, visibility of nurses’ contribution to patient’s health outcomes becomes distinct. In this way, nurses can justify the claim that nursing is a science and an independent profession [7, 8].

Nurses are the largest group of health professionals in all countries. Nursing care quality is closely related to a health care system’s effectiveness. In order to achieve the quality of health care service, quality of nursing care is the key element and to fill this demand application of the nursing process has a significant role, but, in practice, application of the nursing process is not well developed [9].

The gap between research evidence and clinical practice is one of the most persistent problems in the provision of quality healthcare. Approximately 30–40% of patients do not receive health care according to current scientific evidence and some patients receive unnecessary or harmful care [10].

Since nurses are the key caregivers in hospitals, they can significantly influence the quality of care provided and, ultimately, treatment and patient outcomes. Despite their knowledge of the nursing process, certain factors limited the ability of nurses to implement it in their daily practice, including lack of time, high patient volume, and high patient turnover. Despite these hurdles, the daily application of the nursing process is characterized by the scientific background of the professionals involved since it requires Knowledge and provides individualized human assistance [8].

Though application of nursing process is running as a standard of care for many developed countries practically it faces many challenges. Despite there is no strong commitment on the implementation of nursing process in many hospitals in our country the problem is rampant. As we know many studies has been conducted and they identified different factors for the implementation of nursing process. Irrespective of many identified factors that impend nursing process implementation, the purpose of the current survey was to evaluate the implementation of the nursing process at three randomly selected governmental hospitals found in Amhara Region North West Ethiopia.

## Main text

### Method

The study was conducted in three randomly selected governmental hospitals found in Amhara Regional state, North Western Ethiopia, Namely Felege Hiwote Comprehensive Referral Hospital, Debre Tabor General Hospital and Finoteselam General Hospital. The study was conducted from February 30 to March 30/2017.

### Study design

Hospital-based descriptive and retrospective study design was used and the nursing process registration in inpatient records was also checked to inquire all pertinent information regarding on the implementation of nursing process.

### Population

#### Source population

All patient documents’ found in the selected hospital’s record unit and in the nursing station room.

#### Study population

All sampled patient documents’ found in the selected hospital’s record unit and nursing station room having Medical Registration Number (MRN) in the last 6 months.

#### Inclusion and exclusion criteria

All cases from medical, surgical and orthopedics wards were included whereas cases from the outpatient department were not included.

#### Sample size determination

Sample size (n) is determined using a single proportion formula using proportion (p) of nursing process implemented 32.7% from a study conducted in Arbaminch General Hospital, South Ethiopia [11] level of precision (d) 0.05 at 95% confidence interval (Zα/2) that gives 338 cases to be included.

#### Sampling technique

In order to select a representative sample from each hospital, the total number of inpatients in the last 6 months were obtained and then samples were proportionally allocated for each hospital. Based on this, 160 patient’s documents were seen from Felege Hiwote Comprehensive Referral Hospital, 110 from Debre Tabor General Hospital and 68 were seen from Finote Selam General Hospital. Then systematic random sampling using patients MRN was used to select eligible documents.

### Data collection procedures

#### Instrument for data collection

Socio-demographic information that includes: age, sex, educational status, marital status, occupation, ethnicity, previous hospitalization, length of hospitalization were included. Nursing process implementation checklist was prepared in English language to assess the documentation which was done by nurse professionals in the three hospitals.

#### Personnel for data collection

A total of 3 personnel were involved for data collection process and one BSc nurse was assigned to supervise the overall data collection process. All data collectors were oriented for a half day about the instrument and the data collection process.

#### Data quality control

Before conducting the actual data collection, pretest was done on 5% of the total sample size in Debre Markos Hospital. The collected data were reviewed and checked for completeness and relevance by the supervisor. Incomplete questionnaires were returned to the data collectors on the following day for the correction by revisiting the patient’s document.

#### Dependent variable

Implementation of nursing process.

#### Independent variable

- Socio-demographic factors
- Nursing care provided.

#### Operational definition and definition of terms

**Table:** 

Term	Definition
Nursing process	A deliberate problem-solving approach for meeting people’s health care and nursing needs; common components are assessment, diagnosis, planning, implementation, and evaluation
Assessment	The systematic collection of data to determine the patient’s health status and any actual or potential health problems
Nursing diagnoses	Actual or potential health problems that can be managed by independent nursing interventions
Planning	Development of goals and outcomes, as well as a plan of care designed to assist the patient in resolving the diagnosed problems and achieving the identified goals and desired outcomes
Implementation	Actualization or carrying out of the plan of care through nursing interventions
Evaluation	Determination of the patient’s responses to the nursing interventions and the extent to which the outcomes have been achieved.
Nursing process implemented	Hospitals that document all the components of nursing process in the patient file

#### Data procedures and analysis

The data were edited, coded and entered into Epi-Data version 3.1 and exported to IBM SPSS Statistics Version 20 for analysis. Results of the data analysis were presented in the form of descriptive statistics which included mean, standard deviation and percentages. The results were summarized and presented by tables, charts, and graphs.

### Result

Among the reviewed documents 190 (56.2%) were male and 148 (43.8%) were female. Two hundred twenty-four (66.3%) were married whereas 9 (2.7%) were widowed. Three hundred seven (90.8%) were orthodox Christian while 2 (0.6%) were protestant in their religion. Regarding educational status 138 (40.8%) were illiterate were as 45 (13.3%) were completed college and above. Concerning to occupation 75 (22.2%) were housewife whereas 2 (0.6%) were Non-governmental Organizations (NGOs) worker. One hundred eighty-nine (55.9%) were living in the rural part whereas 149 (44.1%) were living in urban areas (Table 1). The minimum age of the study participants was 18 and the maximum age was 88, the average hospital stay was almost 8 days.

**Table 1:** Background characteristics of study participants, Amhara Region Public Hospitals, North West Ethiopia, 2017

Background variables	n	%
Socio-demographic characteristics		
Sex		
Male	190	56.2
Female	148	43.8
Current marital status		
Married	224	66.3
Single	88	26.0
Divorced	17	5.0
Widowed	9	2.7
Religion		
Orthodox	307	90.8
Muslim	25	7.4
Catholic	4	1.2
Protestant	2	0.6
Educational status		
Illiterate	138	40.8
Read and write only	53	15.7
1–4 grade	24	7.1
5–8 grade	37	10.9
9–12 grade	41	12.1
College and above	45	13.3
Occupation		
Housewife	75	22.2
Merchant	51	15.1
Governmental employee	40	11.8
NGOs	2	0.6
Others^a^	170	50.2
Residence		
Urban	149	44.1
Rural	189	55.9
Total	338	100

^a^Daily laborer, student, driver

### Overall implementation of nursing process

From the total 338 reviewed documents, 264 (78.1%) have a nursing process attached with the patient’s profile/file, 107 (31.7%) had no nursing diagnosis, 185 (54.7%) of nurses stated their plan of care based on priority, 173 (51.2%) of nurses did not document their interventions based on plan and 179 (53.0%) of nurses did not evaluat*e* their interventions.

From the total 160 reviewed documents at Felege Hiwote Referral Hospital 100 (62.5%) of patient’s documents had nursing process attached with their file, from 110 reviewed documents at Debretabor General Hospital 99 (90%) of patient’s documents had nursing process attached with their file. Sixty eight documents were reviewed at Finoteselam General Hospital; 65 (95.6%) of patient’s documents had nursing process attached with their file.

Regarding documenting the nursing diagnosis; among the total reviewed 160 documents at Felege Hiwote Referral Hospital 95 (59.4%) had nursing diagnosis whereas from the total 68 reviewed documents at Finoteselam General Hospital 54 (79.4%) had a nursing diagnosis.

Concerning to nursing intervention; 65 (40.6%) of reviewed documents at Felege Hiwote Referral Hospital had a written nursing intervention whereas 63 (57.3%) of reviewed documents at Debretabor General Hospital had a written nursing intervention. Sixty-one (55.5%) documents reviewed at Debretabor General Hospital had evaluated their nursing interventions whereas 66 (41.3%) of reviewed documents at Felege Hiwote Referral Hospital had evaluated their nursing interventions. Overall discrepancies have been found in the implementation of nursing process among the three hospitals (Table 2).

**Table 2:** Implementation of nursing process by hospitals, North West Ethiopia, 2017

Nursing process implementation variables	Felege Hiwote Referral Hospital				Debre Tabor General Hospital				Finote Selam General Hospital			
	Yes		No		Yes		No		Yes		No	
	n	%	n	%	n	%	n	%	n	%	n	%
Is there nursing process attached with the patients’ file?	100	62.5	60	37.5	99	90	11	10	65	95.6	3	4.4
Is there a clearly stated nursing diagnosis?	95	59.4	65	40.6	82	74.5	28	25.5	54	79.4	14	20.6
Does the plan of care stated based on priority?	67	41.8	93	58.2	70	63.6	40	36.4	48	70.6	20	29.4
Does the nursing intervention well documented based on their plan?	65	40.6	95	59.4	63	57.3	47	42.7	37	54.4	31	45.6
Do nurses evaluate their intervention?	66	41.3	94	58.7	61	55.5	49	44.5	32	47.1	36	52.9

**Fig. 1:**
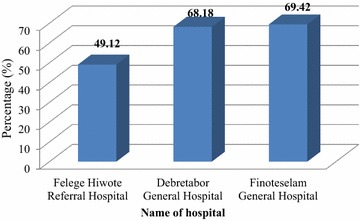
Over-all implementation of nursing process among hospitals in Amhara Region, North West Ethiopia, 2017

The overall implementation of nursing process of the two hospitals namely Debretabor General Hospital and Finoteselam General Hospital had almost similar implementation where as Felege Hiwot Referal Hospital’s performance in implementing the nursing process was low in comparision with the aformentioned two hospitals (Fig. 1).

### Discussion

The aim of this survey was to evaluate the implementation status of nursing process at three governmental hospitals of Amhara Region, North West Ethiopia. Ethiopia as one of the developing country faced limited resource to implement different health and health-related strategies.

Many studies on the implementation of Nursing Process and associated factors among nurses have been done here in Ethiopia and as well in abroad. But almost all of the study subjects were nurses themselves and as we know these will lead a social anticipated bias that may overestimate the finding. But the current survey may overcome this drawback since we are collecting the data after reviewing patient documents (document review). And the finding of the current survey was low which was 47.0%.

Utilization of the nursing process in many low and middle-income countries has been a challenge [7]. All of the respondents said that they did not use the nursing process during provision of care to their patients at the time of the study. The majority (75%) of the respondent said that the nurse to patient ratio was not optimal to apply the nursing process [9]. The main discrepancy here is that before 2015 the implementation of nursing process was not a big deal in many hospitals rather there was a format which was prepared by the federal ministry of health which was different from nursing process. A study was done in Nigeria also revealed that although the trained nurses at the hospital had good theoretical knowledge of the nursing process, they did not apply it in the care of their patients [12].

Hospital nursing services managers (matrons) shall arrange and facilitate case presentations by the nursing staffs which focus on documentation and updates on nursing process.

## Limitation

The current study is a survey of secondary data from a nursing process implementation and this method does not provide the cause effect relationship.

## Abbreviations

NP: Nursing process
MRN: Medical Registration Number
NGOs: Non-governmental Organizations
RERC: Research Ethical Review Committee
